# A new quantification method for mechanical dyssynchrony with three-dimensional echocardiography; segmental time and volume loss for prediction of response to cardiac resynchronisation therapy

**DOI:** 10.1007/s10554-012-0019-3

**Published:** 2012-02-03

**Authors:** J. A. van der Heide, M. F. A. Aly, S. A. Kleijn, J. van Dijk, O. Kamp

**Affiliations:** 1 Department of Cardiology, Institute for Cardiovascular Research (IcaR-VU), VU Medical Center, VU University Medical Center, De Boelelaan 1117, 1081 HV Amsterdam, The Netherlands; 2 Department of Cardiology, Institute for Cardiovascular Research (IcaR-VU), VU Medical Center, VU University Medical Center, P.O. Box 7057, 1007 MB Amsterdam, The Netherlands

**Keywords:** Cardiac resynchronisation therapy, Three-dimensional echocardiography, Left ventricle, Dyssynchrony

## Abstract

A novel method to assess left ventricular (LV) mechanical dyssynchrony using three-dimensional echocardiography (3DE) and semi-automated border detection was investigated, which might be superior in prediction of response to cardiac resynchronisation therapy (CRT) compared to traditional measures that rely solely on segmental time-to-contraction. Twenty-eight heart failure patients underwent real-time 3DE before CRT and at 6–12 months follow-up. Analysis of 3DE was performed using TomTec Research-Arena software featuring semi-automated endocardial border detection. The following echocardiographic parameters were calculated in a 16-segment model: areas under segmental time-volume-curves (STV); delay between contraction of the earliest and latest segment (L-E); and standard deviation of segmental time-to-contraction (SDI). Response to CRT was defined as ≥10% decrease in LV end-systolic volume at follow-up. Baseline Pre-STV had a higher sensitivity than SDI for prediction of response (94 vs 67%, respectively), with equal specificity (78%) and a higher area under receiver operator characteristic curve. In contrast, L-E had a sensitivity of 83% and a specificity of 56%. Using 3DE, methods that combine segmental time-to-contraction with segmental contractility might improve LV dyssynchrony assessment compared to traditional methods based on segmental time-to-contraction alone. Pre-STV might be a better predictor of response to CRT than SDI.

## Introduction

Heart failure remains a complex clinical syndrome. While optimal medical therapy improves survival, cardiac resynchronisation therapy (CRT) has been proven as an effective adjuvant treatment for patients with moderate-to-severe systolic heart failure and wide QRS on optimal medical therapy [1]. However, despite these impressive results, approximately one-third of patients fail to improve after CRT [2]. While the precise mechanisms responsible for the benefits from CRT are not fully understood, the reduction of ventricular dyssynchrony following CRT has been shown to produce favourable acute haemodynamic and neurohormonal changes as well as ventricular remodelling with concomitant symptomatic improvement [2]. Consequently, different imaging-based methods for the assessment of myocardial dyssynchrony have been intensively investigated to improve the prediction of response to CRT [3]. The ideal parameter for the selection of patients for CRT should be simple and practical, and it should not require an elaborate off-line analysis with low reproducibility [4].

Most echocardiographic methods to assess left ventricular (LV) mechanical dyssynchrony rely on timing differences of contraction of individual LV segments, for example Tissue Doppler Imaging (TDI) [5, 6] and three-dimensional echocardiography (3DE) [3, 7–9]. Although poor correlation between 3DE and TDI has been observed [10–12], these methods have been found able to predict response to CRT in single-center studies [6, 10, 11]. By contrast, however, the multi-center PROSPECT-study [13] thoroughly evaluated a wide range of echocardiographic parameters in response to CRT, with disappointing results. Possibly, low reproducibility of various parameters reported in this study might have caused their low predictive values. Time difference between lateral and septal peak systolic wall velocity measured by TDI Doppler achieved an area under receiver operator characteristic (ROC) curve of only 0.61 for the ability to predict LV end-systolic volume (ESV) reduction; reproducibility of this parameter was not reported. The abovementioned methods are based on measurement of timing of contraction of individual LV segments, but it appears that additional factors should be assessed in prediction of response to CRT. We feel that hitherto, segmental differences in contractility (as opposed to segmental differences in timing) have been consistently overlooked.

Assessment of LV mechanical dyssynchrony by 3DE is based on semi-automatic endocardial border detection [14–16]. These systems use segmental time-volume curves which give detailed regional information on LV function. When using software featuring segmental time-volume curves, we observed two distinct patterns: segments with a long contraction delay with preserved (albeit delayed) endocardial inward motion, and segments with a similarly long contraction delay but with decreased endocardial inward motion. When these latter segments are resynchronized by CRT, LV function might fail to improve because irrespective whether those severely hypokinetic segments contract synchronously or not, they contribute very little to global LV function and resynchronisation may not cause significant improvement.

In the present study, we investigate the novel measure of integrating area under time-volume curves for assessment of LV mechanical dyssynchrony in heart failure patients. We hypothesize that this approach, in comparison to traditional 3DE parameters of LV mechanical dyssynchrony, may be of additional value in prediction of response to CRT.

## Methods

A total of 28 heart failure patients were included, with a good echocardiographic apical window and who were eligible for CRT by traditional criteria (ejection fraction [EF] ≤35%, NYHA class III-IV despite optimal medical therapy and a QRS complex ≥120 ms. Exclusion criteria were atrial fibrillation, poor echocardiographic image quality and more than moderate mitral regurgitation. Before permanent pacemaker implantation, NYHA class was assessed, 6-min walk test was performed and a Minnesota living with heart failure questionnaire score was obtained. Furthermore, patients underwent real-time 3DE. These measurements were repeated at 6–12 months follow-up. Off-line, 3DE analysis was performed. Predictive values of response to CRT of baseline echocardiographic parameters were calculated as described below.

### Real-time 3DE

For 3DE, a Philips iE 33 ultrasound machine in combination with an X-4 matrix array transducer was used, according to a method described earlier [15]. Patients underwent 3DE in left lateral decubitus position. Recordings were made from the apical window and care was taken to encompass the entire LV in the data set. Gain and compress settings were adjusted to yield optimal endocardial border definition and depth was adjusted to include aortic and mitral valves at the highest possible frame rate. During a short breath-hold, a full volume consisting of 4 partial volumes was acquired. When during acquisition an extrasystole occurred or a motion artifact was observed, the acquisition was discarded. Acquisitions were stored on CD-ROM for later analysis.

### Image analysis

Off-line, TomTec Research-Arena 3.0 software featuring semi-automated endocardial border detection was used. The software loads an echo acquisition and shows a 2-chamber, 3-chamber, and 4-chamber view on screen, which can be manually aligned with the LV central axis if necessary. The operator traces endocardial borders in all three views, in end-diastole and end-systole. The software then automatically detects the endocardial borders in 3 dimensions, throughout the cardiac cycle, and divides the LV in a 16-segment model. It calculates LV volumes and EF as well as systolic dyssynchrony index (SDI), defined as the standard deviation of times from QRS complex tot minimal segmental volume; and latest minus earliest segment (L-E), which is defined as the time between earliest and latest segment to reach minimal volume. Both SDI and L-E are expressed as a percentage of RR-interval.

### Pre- and Post-STV

**Fig. 1:**
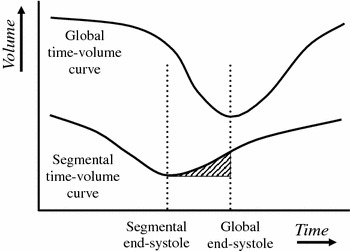
The time-volume loss concept. A global and a segmental time-volume curve are shown. Clearly, the segment shows early contraction. Segmental end-systolic time and volume are measured. Furthermore, time between global and segmental end-systole are measured, and segmental volume difference between global and segmental end-systole. Pre-STV is defined as area under time-volume curve as indicated by the barred area. Thus, a segment with a large time difference but with low contractility will be assigned a low value. Only segments with both large time differences and high contractility, that are expected to yield the most improvement in CRT, will be assigned high values. Pre-STV is the sum of all segments that reach minimal segmental volume before global end-systole

Furthermore, the software calculates Pre-systolic time-volume loss (Pre-STV), which is defined by the area under time-volume curve, as shown in Fig. 1. In calculation of Pre-STV, global end-systolic time is defined as the moment of minimal LV volume. However, the moment of minimal LV volume is the moment that the *sum of all* segmental volumes is at its minimum, which does not necessarily mean that at that moment, each *individual* segmental volume is at its minimum. On the contrary; as is evident from the concept of SDI, each segment reaches its individual minimal volume at different times, which are further spread apart as dyssynchrony worsens. In measurement of Pre-STV, not only timing of minimal volume, but also segmental volume difference between the moments of global and individual minimal volumes is assessed. An individual segment that has reached minimal volume shortly before global end-systole, will be in an early phase of relaxation at global end-systole, with a slight increase in segmental volume. Pre-STV is not only dependent of timing difference but also of this volume difference; the area-under-curve, indicated in Fig. 1, is measured. Pre-STV is the sum of values of all segments that reach minimal segmental volume before global end-systole. Because the X-axis of a time-volume curve is expressed in percentage of RR-interval and the Y-axis is expressed in ml, Pre-STV is expressed in ml × percentage of RR-interval.

The example given in Fig. 1 shows a segment with early segmental minimal volume, but there is of course also the possibility of late segmental minimal volume. These segments are calculated in a similar fashion; the value is called Post-STV. Finally, (total) STV is defined as the sum of Pre- and Post-STV.

The rationale of Pre- and Post-STV is that they do not only assess time to contraction but also segmental LV function. This way, values of segments that show dyssynchrony in combination with decreased contractility are low, which might be of additional value in prediction of response to CRT. Pre- and Post-STV might serve as a correction of SDI for regional contractility.

### Follow-up

After 6–12 months, patients returned for repeat 3DE, all parameters mentioned above were measured and compared to their values at baseline. Response was defined as a ≥10% reduction in ESV compared to baseline and predictive value for response of the abovementioned parameters was calculated [7, 17].

### Reproducibility

For assessment of reproducibility, a subgroup of 15 patients was analyzed by two independent observers. The first observer then repeated analysis after 3 weeks.

### Statistics

Values were expressed as average ± standard deviation, in ml and ms or percentage of the R–R interval. Comparison of values at baseline and follow-up of responders and non-responders was performed using the student’s T-test. Predictive value of response was calculated by ROC curve calculation and by sensitivity, specificity, negative (NPV) and positive predictive value (PPV) calculation. A *p*-value < 0.05 was considered statistically significant. Reproducibility was assessed using Bland–Altman analysis.

## Results

For baseline patient characteristics, see Table 1. There was a majority of males and a high percentage of patients with ischemic heart disease in our patient group. Beta-blocker use was relatively low.

**Table 1:** Baseline clinical patient characteristics

Age	65 ± 10 years
Males/females	21/7
Ischemic heart disease	63%
ACEI/ARB use	79%
Beta-blocker use	61%
Diuretic use	86%
Potassium-sparing diuretic use	54%

Echocardiographic and clinical parameters at baseline and at follow-up are given in Table 2. All patients underwent CRT and follow-up was 100%. There were 8 non-responders and 20 responders in this patient cohort.

**Table 2:** Three-dimensional echocardiography at baseline and at follow-up

Parameter	Baseline				Follow-up				Baseline versus Follow-up		
	All patients	Responders	Non-responders	*p*, responders versus non-responders	All patients	Responders	Non-responders	*p*, responders versus non-responders	*p*, all patients	*p*, responders	*p*, non-responders
EDV (ml)	188 ± 55	195 ± 45	176 ± 60	0.53	168 ± 57	148 ± 50	192 ± 56	0.03	0.04	<0.01	0.05
ESV (ml)	152 ± 52	155 ± 43	144 ± 58	0.33	130 ± 53	110 ± 40	155 ± 57	<0.01*	0.01	<0.01	0.07
EF (%)	20 ± 10	19 ± 8	20 ± 11	0.84	24 ± 10	27 ± 8	20 ± 12	0.07	0.01	<0.01	0.95
L-E (%)	37 ± 18	41 ± 18	25 ± 10	0.01	30 ± 13	29 ± 14	34 ± 12	0.39	0.21	0.02	0.11
SDI (%)	10.8 ± 5.5	12.6 ± 5.7	7.4 ± 3.3	0.01	8.8 ± 3.8	8.5 ± 4.0	9.5 ± 3.4	0.57	0.07	<0.01	0.20
Pre-STV (ml%)	36.0 ± 47.1	51.4 ± 51.2	5.2 ± 6.8	<0.01	9.3 ± 19.9	10.2 ± 23.6	7.3 ± 6.9	0.74	0.01	<0.01	0.28
Post-STV (ml%)	11.2 ± 26.7	12.9 ± 32.7	7.6 ± 5.6	0.51	14.7 ± 18.3	15.1 ± 20.3	13.7 ± 13.8	0.86	0.52	0.78	0.12
BNP (ng/l)	1799 ± 1560	1499 ± 1356	2546 ± 1910	0.26	1467 ± 1465	1065 ± 1058	2407 ± 1932	0.06	0.33	0.40	0.54
NYHA Class	2.9 ± 0.3	2.9 ± 0.3	3.0 ± 0.0	0.43	2.3 ± 0.5	2.3 ± 0.5	2.4 ± 0.5	0.61	<0.01	<0.01	0.03
6 MWT	426 ± 105	425 ± 114	430 ± 86	0.90	474 ± 114	484 ± 114	452 ± 120	0.55	<0.01	0.01	0.15
QOL score	31 ± 16	28 ± 15	37 ± 17	0.27	20 ± 17	18 ± 15	24 ± 21	0.40	<0.01	<0.01	0.04

*EDV* End-diastolic volume, *ESV* End-systolic volume, *EF* Ejection fraction, *L*-*E* Latest minus earliest segment, *SDI* Systolic dyssynchrony index, *Pre*-*STV* Pre-systolic time-volume loss, *Post*-*STV* Post-systolic time-volume loss, *BNP* Brain natriuretic peptide, *6 MWT* Six-min walk test, *QOL score* Quality of life score

* *p* value significant by definition

Responders demonstrated a significant decrease in LV volumes (by definition in ESV) and increase in EF whereas non-responders demonstrated a non-significant increase in LV volumes and no change in EF.

L-E at baseline was significantly higher in responders. At follow-up, it showed a significant decrease in responders and no change in non-responders. SDI showed a similar pattern. There was a large and significant difference in Pre-STV at baseline between responders and non-responders. At follow-up, Pre-STV decreased significantly in responders with no change in non-responders. Post-STV at baseline was not significantly different in non-responders and in responders. No significant changes were observed during follow-up. There was a large and significant difference between Pre- and Post-STV at baseline (*p* < 0.01).

Of the clinical parameters, the decrease in BNP (brain natriuretic peptide) at follow-up reached borderline statistical significance, and there was no significant difference between baseline and follow-up. NYHA class and QOL (Minnesota Living with Heart Failure questionnaire) score improved equally in responders and non-responders whereas 6-MWT (six-min walk test) increased significantly only in responders.

Predictive values for response to CRT of selected echocardiographic parameters are given in Table 3. Pre-STV had the highest sensitivity and specificity and NPV and PPV of all parameters except for SDI, which had equal specificity. Area under ROC curve of Pre-STV was not significantly higher than that of SDI (*p* = 0.38). L-E, SDI and Pre-STV showed significant correlation to reverse remodeling, defined as ESV change at follow-up, with the highest correlation for Pre-STV. Area under ROC curve of Post-STV was not significantly higher than 0.5. There was no correlation between Post-STV and reverse remodeling.

**Table 3:** Predictive values of response to CRT

Parameter	Cut-off value (%)	Sensitivity (%)	Specificity (%)	NPV (%)	PPV (%)	Area under ROC curve (95% CI)	Correlation to Δ-ESV	
							*r*	*p*
L-E	24	83	56	78	89	0.76 (0.58–0.94)	−0.43	0.02
SDI	8.2	67	78	54	86	0.81 (0.64–0.98)	−0.40	0.04
Pre-STV	9.1 ml	94	78	88	89	0.93 (0.82–1.00)	−0.55	<0.01
Post-STV	15.3 ml	22	89	36	80	0.37 (0.14–0.60)	−0.30	0.14

*L*-*E* Latest minus earliest segment, *SDI* Systolic dyssynchrony index, *Pre*-*STV* Pre-systolic time-volume loss, *Post*-*STV* Post-systolic time-volume loss, *NPV* Negative Predictive Value, *PPV* Positive Predictive Value, *Δ*-*ESV* end-systolic volume change at follow-up

**Fig. 2:**
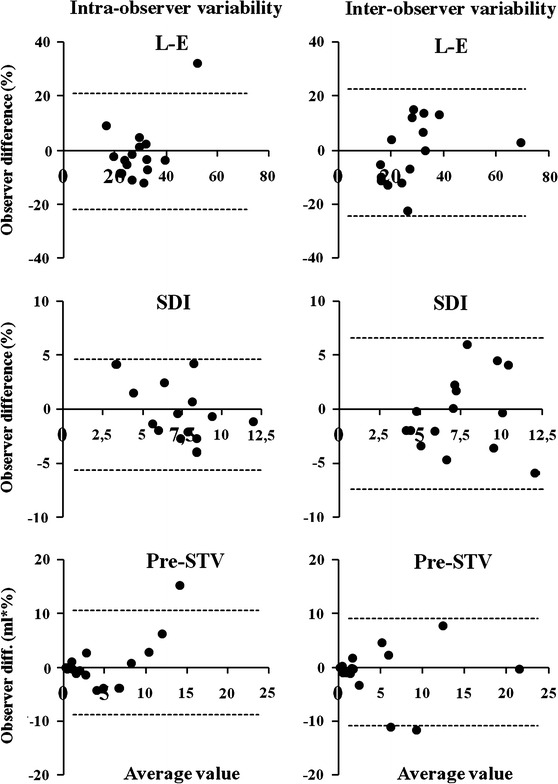
Reproducibility of LV dyssynchrony parameters. Intra-observer variability Inter-observer variability. On the horizontal axis, average value of 2 observations are shown, and on the vertical axis, difference between observations. The dashed bars represent 95% confidence intervals. *L-E* Latest minus earliest segment, *SDI* Systolic dyssynchrony index, *Pre-STV* Pre-systolic time-volume loss

Bland–Altman plots of reproducibility of SDI, L-E and Pre-STV are shown in Fig. 2. On close examination of the Pre-STV plots, it appears that low Pre-STV measurements are very reproducible. However, we did not perform statistical analysis on this specific issue due to the low number of patients.

## Discussion

The present study is the first to demonstrate the capacity of 3DE to assess mechanical dyssynchrony combined with segmental contractility to predict response to CRT in heart failure patients.

While CRT is a well-established therapeutic approach in moderated-to-severe heart failure patients with reduced LVEF (≤35%) and wide QRS (≥120 ms) [1], one-third of patients fail to improve clinically or according to remodelling parameters such as ESV after the implantation of a CRT device. The QRS duration only assesses electrical dyssynchrony and it is use for a selection of patients eligible for CRT has been questioned [18–20].

Several studies focused on echocardiographic indices of mechanical dyssynchrony in heart failure patients to predict response to CRT, defined as a reduction in ESV at follow-up. In single-center studies, reasonable predictive values were reported for very distinct echocardiographic parameters: time difference between 4 basal segments, and standard deviations of time with TDI (in a 12-segment model) and 3DE (in 16- or 17-segment model) [10–12, 15, 21–27]. However, different echocardiographic techniques for assessment of cardiac mechanical dyssynchrony are not interchangeable and conflicting reports exist on their correlations [9, 15, 16, 28]. In the PROSPECT study [13], the only measure that demonstrated predictive value was time difference between lateral and septal peak systolic wall velocity as measured by TDI; it achieved an area under ROC curve of only 0.61 (*p* = 0.01). However, new approaches like the one we tested might help in finding a simple, robust parameter to add to the current criteria proposed by guidelines to decide upon or not to propose to a patient a CRT device.

The present study investigates cardiac mechanical dyssynchrony fundamentally differently from all previous studies, and is proof-of-concept of assessment of mechanical dyssynchrony using parameters that are not only dependent of segmental timing, but also on (decreased) segmental endocardial inward motion. It is conceivable that non-responders with high SDIs might have dyssynchrony mainly in hypokinetic segments. By correcting SDI for regional contractility, STV can provide more comprehensive information on the severity of mechanical dyssynchrony with the ultimate goal of improving the prediction of response to CRT.

### STV and SDI

Correlation between STV and SDI is moderate because STV and SDI are fundamentally different parameters. Firstly, STV is calculated by summation of values of all segments, whereas SDI is a standard deviation [15, 16, 26]. The use of a standard deviation requires a large number of normally distributed data, but in SDI there are only 12–17 segments. Presence of a small number of segments with a long delay might cause skewed distribution, impairing robustness of a standard deviation, which is a weakness intrinsic to all standard-deviation based models of mechanical dyssynchrony.

Secondly, since basal segments have higher volumes than the mid- and apical segments, their contribution to the total value of STV is greater than that of mid- and apical segments. This is in contrast to methods that use only timing parameters and where basal, mid- and apical segments contribute equally to the final value.

Finally, reproducibility of SDI is moderate in both the present and in previous studies [7, 8, 15], and as a consequence, SDI values in non-responders and responders vary widely between studies, with partial overlap. SDI in non-responders varies from 3.4 to 7.1%, SDI in responders varies from 9.7 to 16.6% [15, 25–27] and cut-off values between responders and non-responders vary from 5.6 to 10%. It must be emphasised that calculation of all three parameters (L-E, SDI and Pre-STV) was based on the same endocardial borders; reproducibility of SDI and Pre-STV seems to be of a similar magnitude. Close observation of Fig. 2 however, reveals that reproducibility of Pre-STV was very high when values were low (the cluster of dots around the 0.0 mark), which implies that absence of any significant amount of Pre-STV can be measured very reliably.

### Left ventricular dyssynchrony

In agreement with our results regarding the changes observed in Pre-STV, similar changes were found in a TDI study on heart failure patients received CRT, where peak systolic contraction in 6 basal segments was delayed homogeneously to a timing close to that of the latest segment, so that regional variation after CRT was abolished [5]. This seems to contradict the theory that CRT improves LV function by activating late segments earlier. However, this observation may be explained if we consider that the timing of pacing occurs at end-diastole, and dyssynchrony is assessed at end-systole. We were unable to determine whether the decrease in Pre-STV was caused by a leftward shift of the global time-volume curve or by “true” decrease of Pre-STV. In hemodynamic studies on CRT, an increased positive dP/dT was reported [29–31], which might be consistent with the decrease in Pre-STV that we observed. Because events before global end-systole occur during high intraventricular pressure and events after global end-systole occur during low intraventricular pressure, a decrease in Pre-STV might have a larger beneficial influence on myocardial energy efficiency than a decrease in Post-STV.

### Future perspectives

In prediction of response to CRT, the key issue remains accurate and reproducible assessment of left ventricular mechanical dyssynchrony in both contractility and in timing. With minor adaptations in analysis software, (Pre)-STV may be visualised in a polar map, which may aid in optimising biventricular pacemaker lead location. In future studies, time difference between basal, mid- and apical segments [10] and time difference between longitudinal, radial and circumferential contraction [32] should be compared with (Pre) STV or similar measures in prediction of response to CRT. Although speculative, methods that rely on regions-of-interest in combination with tissue Doppler or speckle tracking might obviate the need for endocardial border detection altogether. The present analysis method might be applicable to other imaging modalities as well. Total scar burden, as assessed using contrast-enhanced magnetic resonance imaging, is an important factor influencing response to CRT and may be included in the selection process for CRT candidates [33]. Strain by 3D speckle tracking might be an alternative for the assessment of myocardial necrosis [34] and is also helpful to guide lead position. Systematic evaluation of mitral valve regurgitation [35] might reveal additional information.

### Limitations

The present study was not a randomized, controlled study, but rather a hypothesis generating investigation of a new and still unproven method in a small patient group with a relatively short follow-up period. At present, this method should not be used clinically without further investigation.

Further limitations were in the nature of the software. End-systole was defined as the moment of minimal LV volume. No attention was given to opening or closing of mitral and aortic valves and the isovolumetric relaxation time was not assessed. Although Post-STV is a measure of early diastolic function, we were not able to investigate the events that occur around end-diastole. We could not evaluate if segments in the vicinity of the (postero)lateral pacemaker lead showed the highest Pre- or Post-STV, if they showed the most improvement, or if this had any influence on response to CRT.

## Conclusions

The present method which integrates segmental timing and wall motion might describe LV mechanical dyssynchrony better than methods based solely on timing. In our patient group, Pre-STV might serve as a correction for regional contractility of SDI, and is a better predictor of volume response to CRT than SDI or L-E. Larger scale studies are needed to confirm its prognostic value.

## Abbreviations

3DE: Three-dimensional echocardiography
CRT: Cardiac resynchronsation therapy
EDV: End-diastolic volume
ESV: End-systolic volume
EF: Ejection fraction
L-E: Latest minus earliest segment
LV: Left ventricle
Pre-STV: Pre-systolic time-volume loss
Post-STV: Post-systolic time-volume loss
SDI: Systolic dyssynchrony index
TDI: Tissue Doppler Imaging
